# Efficacy and effects on cardiac function of radiofrequency catheter ablation vs. direct current cardioversion of persistent atrial fibrillation with left ventricular systolic dysfunction

**DOI:** 10.1371/journal.pone.0174510

**Published:** 2017-03-28

**Authors:** Maojing Wang, Shanglang Cai, Wei Ding, Yujie Deng, Qing Zhao

**Affiliations:** 1 Department of Cardiology, The Affiliated Hospital of Qingdao University, Qingdao, China; 2 Ophthalmology Department, Huangdao District People's Hospital, Qingdao, Shandong, China; University of Miami School of Medicine, UNITED STATES

## Abstract

**Objective:**

To evaluate the effect of catheter ablation vs. direct current synchronized cardioversion (DCC) in patients with persistent atrial fibrillation (AF) and left ventricular systolic dysfunction, and to define baseline features of patients that will get more benefit from ablation.

**Methods:**

From July 2013 to October 2014, 97 consecutive single-center patients with persistent AF and symptomatic heart failure (left ventricular ejection fraction (LVEF) <50%) underwent DCC followed by amiodarone (n = 40) or circumferential pulmonary vein isolation (PVI; n = 57) according to patient’s preference were recruited in the study. Post-ablation recurrence was treated with atrial roof and mitral isthmus lines ablation with or without PVI based on restoration or not of pulmonary vein (PV) potential conduction. Study outcomes were 12-month rate of sustained sinus rhythm (SR) and cardiac function. Baseline characteristics were compared between patients with and without cardiac function improvement post ablation.

**Results:**

With similarly distributed characteristics at baseline, ablation (mean 1.8 procedures) relative to DCC yielded significantly higher level of 12-month SR maintenance rate (68.42% vs. 35%, P = 0.001); and better LVEF and New York Heart Association class. with significant effect for DCC only in maintained SR cases. Post ablation LVEF increased (>20% or to over 55%) in 31 (54.39%) patients with worse baseline cardiac function and ventricular rate control.

**Conclusions:**

Catheter ablation relative to cardioversion of persistent AF with symptomatic heart failure yielded better 12-month SR maintenance and cardiac function. Compared with non-responders, patients with improved LVEF post-ablation had poorer ventricular rate control and cardiac function at baseline, suggesting a significant component of tachycardia-induced cardiomyopathy in this group.

## Introduction

Atrial fibrillation (AF) and heart failure (HF) share common etiologies, including hypertension, coronary heart disease, valvular disease, smoking, and sleep apnea, among others[1], and therefore often coexist and influence each other. A rapid and irregular ventricular rate in AF can affect left ventricular systolic function [2] increasing HF incidence by threefold [3], and in turn HF increases AF incidence by six fold [4], with the cross effect increasing mortality [5,6]. Theoretically, AF conversion to sinus rhythm (SR) should improve cardiac function and consequently long-term prognosis of patients with AF with HF. DC synchronized cardioversion (DCC) is commonly used; however, it is often not effective particularly for longer duration AF, and even when successful it has high AF recurrence rates [7] with SR maintenance often requiring use of antiarrhythmic drugs (AADs) with their significant long-term side effects [8]. Because catheter ablation is mainstay strategy for conversion of several forms of AF to SR [9], the present study compared efficacy and effects on cardiac function of DCC and catheter ablation of AF with HF and assessed which patients would benefit most from ablation.

## Materials and methods

### Case selection

In the present prospective single center study conducted at our Department of Cardiology from July 2013 to October 2014 after obtaining protocol approval from the institutional review board, 97 consecutive patients (mean age, 58.39±9.86 years old; 52 males) with persistent atrial fibrillation and left ventricular systolic dysfunction (LVEF <50%) with heart failure symptoms were selected after providing signed informed consent. Patients underwent either DCC or catheter ablation according to their preference. Of the 43 patients who underwent DCC, conversion to SR was achieved in 40 patients who were then treated with amiodarone for SR maintenance; the remaining 3 patients failed DCC and underwent catheter ablation. A total of 57 patients underwent catheter ablation. Persistent atrial fibrillation was defined based on the 2014 AHA/ACC/HRS atrial fibrillation guideline [9]. Study exclusion criteria were: left atrial thrombus confirmed by transesophageal echocardiography; severe heart valve disease; coronary heart disease treated with revascularization within the previous 3 months; reversible causes of atrial fibrillation complicated with heart failure such as hyperthyroidism; alcohol use; and pregnancy. Patients who underwent DCC received warfarin to achieve an INR of 2–3 for over 3 weeks prior to cardioversion, and postoperatively for at least 4 weeks; long-term amiodarone was used to prevent atrial fibrillation recurrence. Patients who underwent catheter ablation received warfarin preoperatively to achieve an INR of 2–3; warfarin was continued postoperatively for at least 3 months and reinstated with AF recurrence. Amiodarone was discontinued 1 month after PVI. Patients were evaluated by esophageal ultrasonography 24 hours prior to ablation to rule out left atrial thrombus.

All the patients had signed a general written informed consent. And ethical approval was given by the medical ethics committee of the Affiliated Hospital of Qingdao University. The study protocol conformed to the ethical guidelines of the 1975 Declaration of Helsinki.

### Mapping and ablation

The ablation strategy for atrial fibrillation was pulmonary vein isolation (PVI). As previously described [10], after puncturing the right internal jugular vein, 10 poles electrophysiology catheters were advanced through the coronary sinus, twice passing the interauricular septum to advance 2 × 8.5F SL1 of Swartz length sheaths to the left atrium by puncturing the interauricular septum. Unfractionated heparin then was administered, and ACT was measured every 30 minutes and maintained in the 300–350 range. Pulmonary vein potential was recorded with a circular mapping catheter 20 or 25 mm in diameter (Lasso, Biosense-Webster, Diamond Bar, CA). PVI was performed with a 3.5 mm ablation catheter (Navi-Star ThermoCool, Biosense-Webster, USA). Under guidance of Carto system, 35 watts of energy were delivered to the pulmonary vein anterior wall using a pump flow rate of 20mL/min; while 30 watts of energy were delivered to the upper and posterior walls using a pump flow rate of 17mL/min. Using temperature controlled discharge mode and a temperature limit of 43°C, discharge duration was 20 seconds or until the potential was reduced by 80% for every ablation point. Ablation procedure was terminated upon achievement of sinus rhythm. For patients remaining in atrial fibrillation, DCC was attempted. Amiodarone or other antiarrhythmic drugs were discontinued 2 months after PVI. For patients with AF recurrence after 3 months post PVI, catheter ablation was repeated with PVI with concurrent left atrium roof line and mitral isthmus line ablation or with only the latter two ablation procedures for patients in whom pulmonary vein potential conduction was or not restored, respectively [11,12]. DCC was attempted in patients who did not convert to SR. For patients with recurrence after 2 ablations, catheter ablation was repeated following the same protocol as the second ablation. Therefore, patients underwent at most 3 catheter ablations.

### Postoperative treatment and follow-up visit

All patients were scheduled for follow-up at 3, 6 and 12 months, and patients were instructed to undergo surface electrocardiography at the local hospital at any suspicion of arrhythmia recurrence. At follow-up visits, surface and 24 hour dynamic electrocardiograms were recorded to document arrhythmia recurrence, defined as presence of any symptomatic atrial fibrillation, atrial flutter and atrial tachycardia or other atrial arrhythmias lasting for over 30 seconds. All patients were evaluated by echocardiography 24 hours after DCC or PVI procedures, and at 6 and 12 months. All patients were evaluated for New York cardiac function classification (NYHA classification) prior and 6 and 12 months after DCC or ablation.

### Statistical analysis

SPSS 19.0 statistical software was used for statistical analysis. Continuous parameters are expressed as mean±standard deviation and were compared using single factor variance analysis, while categorical data are expressed as number (percentage) and were compared using the Chi-square test. Multivariate regression models were used to determine the characteristics that may identify patients that benefit from ablation. P<0.05 was considered statistically significant.

## Results

There was no significant difference in distribution of baseline characteristics between the two study groups (Table 1).

**Table 1:** Comparison of baseline clinical characteristics between the two study groups.

	Ablation group	DCC group	P value
	(n = 57)	(n = 40)	
Male gender	30 (52.6%)	22 (55%)	0.839
Age (years)	58.90±10.10	57.67±9.71	0.489
Left atrial diameter (mm)	46.54±3.52	45.73±3.62	0.268
LVEDD (mm)	55.25±4.19	54.18±3.47	0.367
LVEF (%)	37.93±5.18	39.35±4.89	0.177
NYHA functional class	2.56±0.57	2.63±0.54	0.581
Continuous AF Duration time (months)	20.93±11.27	18.77±8.56	0.312
Hypertension	21 (36.84%)	17 (42.5%)	0.362
Diabetes mellitus	16 (28.07%)	12 (30%)	0.506
Snoring	8 (14.04%)	5 (12.5%)	0.539
Premedication			
Digoxin	17 (29.82%)	11 (27.5%)	0.494
Beta blocker	25 (43.86%)	22 (55%)	0.191
ACE-I or ARB	20 (35.09%)	18 (45%)	0.219
Aldosterone antagonist	12 (21.05%)	9 (22.5%)	0.528
Amiodarone	7 (12.28%)	4 (10%)	0.497
Mean ventricular rate at rest	87.18±13.24	83.25±15.18	0.179
Maximum ventricular rate at activities	127.11±16.65	123.27±22.19	0.333
Medication during FU			
Digoxin	12 (21.05%)	8 (20%)	0.555
Beta blocker	35 (61.40%)	30 (75%)	0.118
ACE-I or ARB	25 (43.86%)	21 (52.5%)	0.264
Aldosterone antagonist	15 (26.32%)	10 (25%)	0.538
Rate of SR at the end of FU	39 (68.42%)	14 (35%)	0.001

Data are shown as mean±SD or n (%).

LVEDD, left ventricular end-diastolic diameter; LVEF, left ventricular ejection fraction; AF, atrial fibrillation; NYHA, New York Heart Association; ACE-inhibitor, angiotensin-converting enzyme inhibitor; ARB, angiotensin receptor blocker; SR, sinus rhythm; FU, follow up.

Catheter ablation was completed in all patients without serious complications in 57 patients. 28 patients underwent a repeat ablation of whom 18 patients (31.6% of the total) had two repeat ablations (total 1.8 ablation procedures per patient).

**Fig 1:**
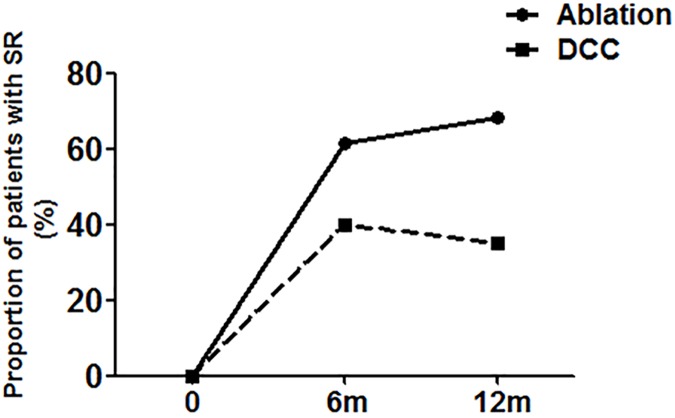
Comparison of proportion of patients with SR at 6 and 12 months between ablation and DCC groups. DCC, direct current cardioversion.

Patients treated with ablation relative to DCC yielded significantly higher level of 6-month SR maintenance rate (61.40% vs. 40%, respectively, P = 0.030); At 12-month follow-up, rate of sinus rhythm maintenance was significantly higher in patients who underwent single PVI (n = 29, 50.88%) or mean 1.8 ablations (n = 39, 68.42%) as compared to those who underwent DCC (n = 14, 35%) (Fig 1).

**Fig 2:**
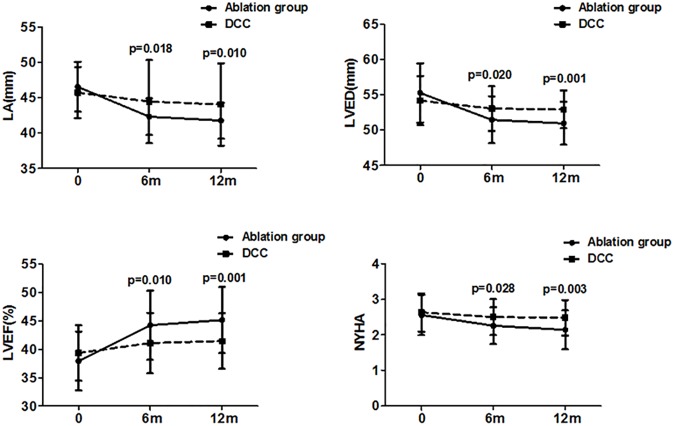
Comparisons of LA, LVEDD, LVEF and NYHA at 6 and 12 months between study groups. LA, left atrial; LVED, left ventricular end-diastolic; LVEF, left ventricular ejection fraction; AF, atrial fibrillation; NYHA, New York Heart Association.

As shown in Table 2, at 6- and 12-month follow-up, LA and LVEDD were lower, LVEF higher, and cardiac function NYHA classification better in ablation but not DCC group relative to baseline; between-group differences for these parameters were significant at both 6- and 12-month follow-up (Fig 2). However, regardless of procedure, patients with maintained sinus rhythm had smaller LA and LVEDD and significant improvement in cardiac function throughout follow-up relative to baseline. However, in patients with non-maintenance of sinus rhythm, sizes of LA and LVEDD and cardiac function had no significantly improvement. (Tables 3 and 4).

**Table 2:** Changes in LA, LVEDD, LVEF and NYHA at 6 and 12 months post ablation or DCC.

	Baseline	Month 6	Month 12	P	p'
Ablation group (n = 57)					
LA (mm)	46.54±3.52	42.33±2.59	41.77±2.56	0.001	0.000
LVEDD (mm)	55.25±4.19	51.46±3.32	50.96±3.04	0.000	0.000
LVEF (%)	37.93±5.18	44.23±6.09	45.16±5.83	0.000	0.000
NYHA	2.56±0.57	2.26±0.52	2.14±0.55	0.004	0.001
DCC group (n = 40)					
LA (mm)	45.73±3.62	44.45±5.88	44.05±5.82	0.246	0.126
LVEDD (mm)	54.18±3.47	53.05±3.15	52.93±2.68	0.133	0.075
LVEF (%)	39.35±4.89	41.08±5.31	41.45±4.90	0.135	0.059
NYHA	2.63±0.54	2.50±0.51	2.48±0.50	0.124	0.078

P: 6 months vs. baseline; p': 12 months vs. baseline.

Data are shown as mean±SD.

LA, left atrial; LVEDD, left ventricular end-diastolic diameter; LVEF, left ventricular ejection fraction; AF, atrial fibrillation; NYHA, New York Heart Association.

**Table 3:** Changes in LA, LVEDD and heart function in patients maintaining or not SR post ablation.

	Baseline	Month 6 SR (n = 35)	P	Baseline	Month 6 NSR (n = 22)	P
LA	44.52±3.14	41.40±2.34	0.000	49.33±1.55	48.29±2.71	0.109
LVEDD	54.76±2.97	50.24±2.49	0.000	55.67±4.80	53.38±4.49	0.094
LVEF	38.00±4.91	44.61±5.52	0.000	37.83±5.64	40.92±5.20	0.055
NYHA	2.61±0.50	2.21±0.55	0.003	2.50±0.66	2.33±0.48	0.323
	Baseline	Month12 SR (n = 39)	P	Baseline	Month 12 NSR (n = 18)	P
LA	45.15±3.28	41.18±2.38	0.000	49.56±1.65	48.51±3.22	0.099
LVEDD	54.05±2.97	50.05±2.48	0.000	55.50±5.60	52.28±4.96	0.076
LVEF	38.00±5.26	45.21±5.45	0.000	37.78±5.65	40.61±6.40	0.169
NYHA	2.67±0.48	2.21±0.57	0.000	2.33±0.69	2.06±0.54	0.186

Data are shown as mean±SD.

SR, sinus rhythm; NSR, non-maintenance of sinus rhythm; LA, left atrial; LVEDD, left ventricular end-diastolic diameter; LVEF, left ventricular ejection fraction; AF, atrial fibrillation; NYHA, New York Heart Association.

**Table 4:** Changes in LA, LVEDD and heart function in patients maintaining or not SR post DCC.

	Baseline	Month 6 SR (n = 16)	P	Baseline	Month 6 NSR (n = 24)	P
LA	43.13±3.88	38.56±3.56	0.002	47.46±2.15	48.38±3.19	0.249
LVEDD	54.94±4.11	52.13±3.42	0.044	53.67±2.96	53.24±2.75	0.179
LVEF	39.06±5.40	43.69±6.36	0.034	39.54±4.63	40.38±5.31	0.289
NYHA	2.69±0.48	2.25±0.58	0.027	2.59±0.58	2.53±0.51	0.601
	Baseline	Month12 SR (n = 14)	P	Baseline	Month12 NSR (n = 26)	P
LA	42.68±4.08	37.08±2.78	0.000	47.12±2.39	47.62±3.35	0.538
LVEDD	53.75±3.80	50.92±2.19	0.030	54.15±3.33	52.96±2.57	0.155
LVEF	41.13±6.95	47.00±5.67	0.024	39.23±4.60	41.23±4.68	0.126
NYHA	2.56±0.63	2.00±0.74	0.039	2.61±0.57	2.54±0.51	0.610

Data are shown as mean±SD.

SR, sinus rhythm; NSR, non-maintenance of sinus rhythm; LA, left atrial; LVEDD, left ventricular end-diastolic diameter; LVEF, left ventricular ejection fraction; AF, atrial fibrillation; NYHA, New York Heart Association.

At 12-month follow-up, 54.39% patients in catheter ablation group showed marked cardiac function improvement (i.e., increased LVEF by >20% or to over 55%) [13], and in comparison to patients without marked cardiac function improvement, baseline rate of average ventricular rate of <80/min at rest was significantly lower (p = 0.026), average ventricular rate and the highest ventricular rate was significantly more rapid (p = 0.023 and 0.008, respectively), and baseline cardiac function was significantly worse (p<0.001) (Table 5).

**Table 5:** Comparison of clinical data between patients with or without cardiac function improvement post ablation.

	Marked improvement in LV (n = 31)	NO (n = 26)	P value
Male gender	18 (58.06%)	12 (46.15%)	0.264
Age (years)	56.87±9.67	60.61±11.03	0.178
Left atrial diameter (mm)	46.35±3.47	46.77±3.64	0.662
LVEDD (mm)	55.39±3.77	54.27±4.14	0.294
LVEF (%)	35.03±4.67	41.38±3.34	0.000
NYHA functional class	2.65±0.63	2.48±0.49	0.264
Continuous AF Duration time (months)	19.65±9.76	22.58±13.05	0.336
Hypertension	12 (38.71%)	9 (34.62%)	0.484
Diabetes mellitus	10 (32.26%)	6 (23.08%)	0.320
Snoring	5 (16.13%)	3(11.54%)	0.458
Premedication			
Digoxin	11 (35.48%)	16 (61.54%)	0.045
Beta blocker	10 (32.23%)	15 (57.69%)	0.048
ACE-I or ARB	7 (22.59%)	13 (50%)	0.030
Aldosterone antagonist	5 (16.13%)	6 (23.08%)	0.371
Amiodarone	4 (12.90%)	3 (11.54%)	0.601
Mean ventricular rate at rest	91.42±14.29	82.65±13.86	0.023
Maximum ventricular rate at moderate exercise	133.35±18.17	120.92±15.74	0.008
Insufficient control of ventricular rate n (%)	21 (67.74%)	10 (38.46%)	0.026
Medication during FU			
Digoxin	8 (25.81%)	4 (15.38%)	0.265
Beta blocker	20 (64.52%)	15 (57.69%)	0.399
ACE-I or ARB	15 (48.39%)	10 (38.46%)	0.315
Aldosterone antagonist	9 (29.03%)	6 (23.08%)	0.420

Data are shown as mean±SD or n (%).

LV, left ventricular; LVEDD, left ventricular end-diastolic diameter; LVEF, left ventricular ejection fraction; AF, atrial fibrillation; NYHA, New York Heart Association; ACE-I, angiotensin-converting enzyme inhibitor; ARB, angiotensin receptor blocker.

Marked improvement in heart function refers to: Improvement in LVEF >20% or to over 55%.

Sufficient control of ventricular rate: Mean ventricular rate at rest <80 beats/min.

Multivariate model analysis showed at the Month 12 the cardiac function improvement in LVEF (P = 0.001) and NYHA (P = 0.003), was independently associated with baseline LVEF≤40% (p = 0.005, hazard ratio (HR)7.632, 95% confidence interval (CI): (1.820–32.007)); baseline Mean ventricular rate >80 beats/min at rest (p = 0.033, HR3.210, 95% CI: (1.405–9.616)); Maximum ventricular rate >110 beats/min at moderate exercise(p = 0.027, HR2.231, 95% CI: (1.389–7.897))(Table 6).

**Table 6:** Variables affecting Marked improvement in LVEF post ablation.

	P value	HR (95% CI)
Male gender	0.397	2.997 (0.237–37.916)
Age≥60(years)	0.471	0.389 (0.030–5.074)
LVEDD≥50(mm)	0.960	1.078 (0.055–21.064)
LVEF≤40%	0.005	7.632 (1.820–32.007)
NYHA functional class≥III	0.614	1.227 (0.554–2.719)
Continuous AF Duration time≥20 (months)	0.423	1.946 (0.382–9.897)
Hypertension	0.855	0.924 (0.396–2.158)
Diabetes mellitus	0.810	1.124 (0.433–2.919)
Snoring	0.394	0.629 (0.216–1.829)
Premedication		
Digoxin	0.210	0.495 (0.164–1.487)
Beta blocker	0.856	1.085 (0.447–2.637)
ACE-I or ARB	0.525	1.384 (0.508–3.774)
Aldosterone antagonist	0.787	1.187 (0.343–4.114)
Amiodarone	0.423	1.946 (0.382–9.897)
Mean ventricular rate at rest>80 beats/min.	0.033	3.210 (1.405–9.616)
Maximum ventricular rate at moderate exercise >110 beats/min.	0.027	2.231 (1.389–7.897)

Data are shown as mean±SD or n (%).

LV, left ventricular; LVEDD, left ventricular end-diastolic diameter; LVEF, left ventricular ejection fraction; AF, atrial fibrillation; NYHA, New York Heart Association; ACE-I, angiotensin-converting enzyme inhibitor; ARB, angiotensin receptor blocker; HR, hazard ratio; CI: confidence interval.

## Discussion

The main findings of the present study of patients with persistent AF with concomitant LVEF<50% were that: 1. catheter ablation as compared to direct current synchronized cardioversion followed by amiodarone is associated with significantly higher 1-year rates of maintained sinus rhythm and improved cardiac function; and 2. patients with poorer ventricular rate control and cardiac function at baseline appear to benefit most from ablation in terms of cardiac function improvement at 1 year.

Drug therapies aimed at maintaining sinus rhythm relative to those aimed at controlling ventricular rate appear to provide no apparent survival benefit to patients with atrial fibrillation and cardiac insufficiency [13,14], because in this setting antiarrhythmic agents have an even lower success rate with side effects that cannot be neglected [14]. In contrast, relative to medications controlling ventricular rate, catheter ablation significantly improves cardiac function of patients with atrial fibrillation and cardiac insufficiency [15–17]. However, risks of catheter ablation are higher in patients with cardiac insufficiency, which renders it clinically relevant to identify which patients would benefit from it.

In previous reports [18,19], approximately half the patients experienced atrial fibrillation recurrence 6 months after cardioversion. In the present study of patients with persistent AF and cardiac insufficiency there was 65% recurrence among DCC patients, which was significantly higher that the approximately third or half of patients who experienced recurrence post single or mean 1.8 ablation procedures. Also, for patients receiving DCC, because of the higher recurrence rate, LVEF and NYHA failed to show improvement in the overall population; however, significant improvement was apparent for patients with sinus rhythm maintenance. It is indicated that the improvement of heart function of patients with persistent AF and symptomatic heart failure due to the maintenance of sinus rhythm. It should be pointed out that the present study excluded patients with severe valvular disease and ischemic cardiomyopathy patients which would not benefit from catheter ablation [20,21]; also, patients studied were relatively young (58.39\9.86 years old), with a relatively short duration of atrial fibrillation. Further studies are warranted to assess and validate the findings in a broader population.

In patients without severe heart valve disease and severe ischemic cardiomyopathy, atrial fibrillation complicated with left ventricular enlargement and left ventricular ejection fraction reduction has often been misdiagnosed as dilated cardiomyopathy. However, in atrial fibrillation, in addition to rapid ventricular rate, the loss of atrial booster pump function and of AV synchrony and R-R irregular interval, might affect left ventricular function [22,23]; moreover, uncontrolled and/or ventricular rate may sometimes lead to tachycardia cardiomyopathy. Clinically, it is very difficult to distinguish a dilated cardiomyopathy resulting in atrial fibrillation from an atrial fibrillation resulting in tachycardia cardiomyopathy. However, after atrial fibrillation has been converted to sinus rhythm, if left ventricular function returns to normal, then tachycardiac cardiomyopathy can be confirmed; otherwise, dilated cardiomyopathy is likely. For atrial fibrillation patients complicated with heart failure and poorly controlled ventricular rate, the likelihood of atrial fibrillation resulting in tachycardia cardiomyopathy may be increased. in the present study, a little over half the patients with persistent AF and heart failure treated with catheter ablation showed improved cardiac function consistent with the presence of reversible tachycardia cardiomyopathy; the other half appeared to present dilated cardiomyopathy.

In the present study, patients who derived cardiac function benefit post ablation, relative to those who did not, showed at baseline lower rate of use of ventricular rate control drugs and ACEIs, lower proportion of sufficient control of ventricular rate, more rapid ventricular rate, and worse baseline cardiac function. cardiac function improvement was independently associated with worse baseline LVEF and lower proportion of sufficient control of ventricular rate.

## Limitations

The present study is limited by its single center design with a relatively small sample size which excluded patients with persistent atrial fibrillation and heart failure at higher risk for complications associated with catheter ablation [24], such as those with longer duration of AF, older patients and those with severe valvular disease and ischemic cardiomyopathy [25]. Further larger, randomized studies including a broader patient base are warranted.

## Conclusions

For patients with persistent atrial fibrillation complicated with symptomatic left ventricular systolic dysfunction, catheter ablation appears more efficacious than cardioversion in terms of sinus rhythm maintenance and cardiac function improvement. Patients with atrial fibrillation, left ventricular enlargement and heart failure, may have dilated or tachycardia cardiomyopathy, with the latter benefitting most in terms of cardiac function improvement from catheter ablation and displaying at baseline more inadequately controlled ventricular rate and cardiac function. Thus, compared with a group of patients preferring cardioversion for catheter ablation, catheter ablation seems to increase the proportion of patients in sinus rhythm and improve cardiac function after 12 months.

## Supporting information

S1 Table Baseline clinical characteristics. (XLS)

S2 Table Changes in LA, LVED and heart function in patients maintaining or not SR post ablation. (XLS)

S3 Table patients with or without cardiac function improvement post ablation. (XLSX)

S1 Fig ethics committee approval 01. (TIF)

S2 Fig ethics committee approval 02. (TIF)
