# Supplementary figures and images for: Efficacy and effects on cardiac function of radiofrequency catheter ablation vs. direct current cardioversion of persistent atrial fibrillation with left ventricular systolic dysfunction
Source: PLoS One. 2017 Mar 28;12(3):e0174510. doi: 10.1371/journal.pone.0174510 (PMC5370131; doi:10.1371/journal.pone.0174510)

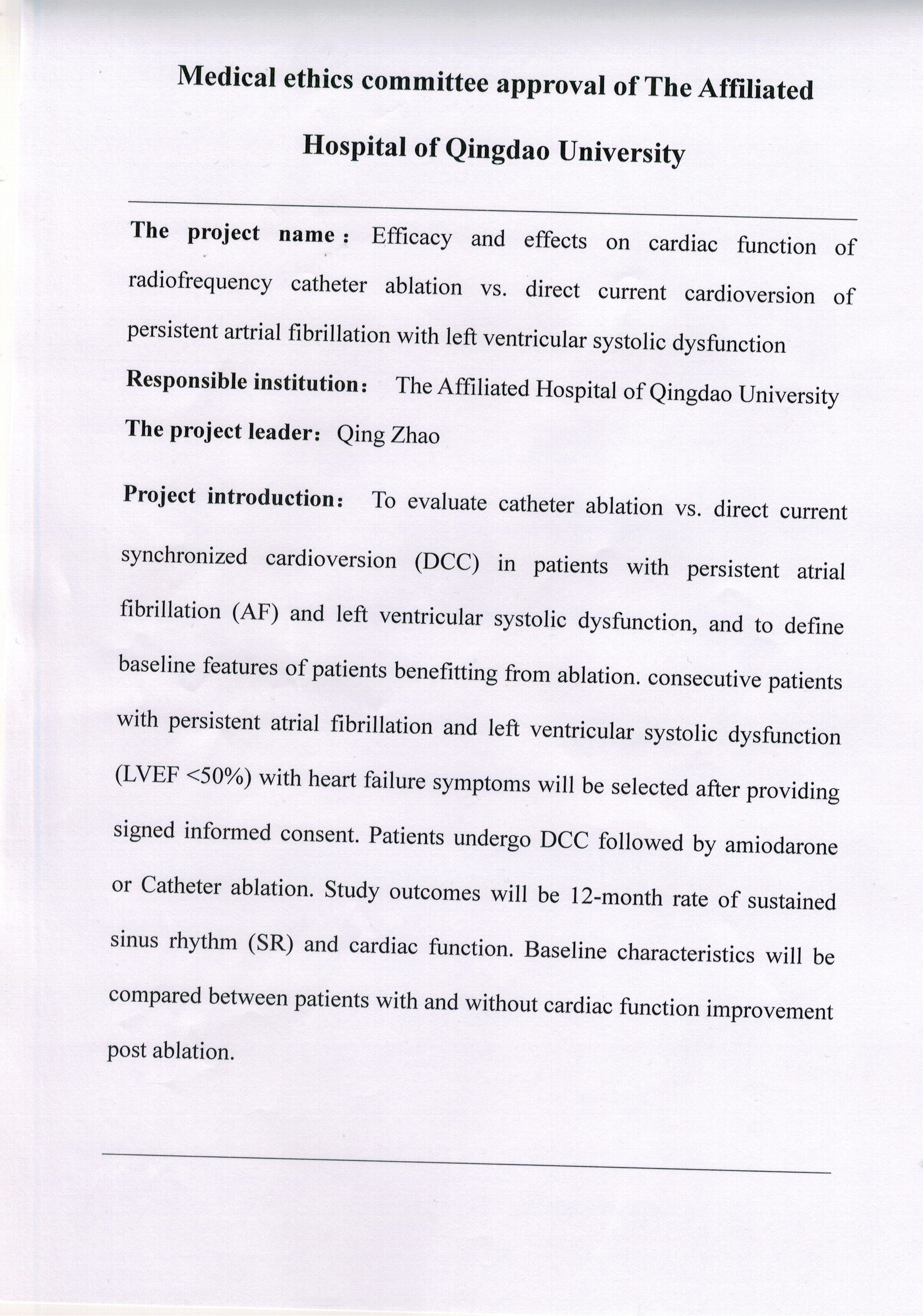

Supplement: S1 Fig — (TIF) [file pone.0174510.s004.tif]

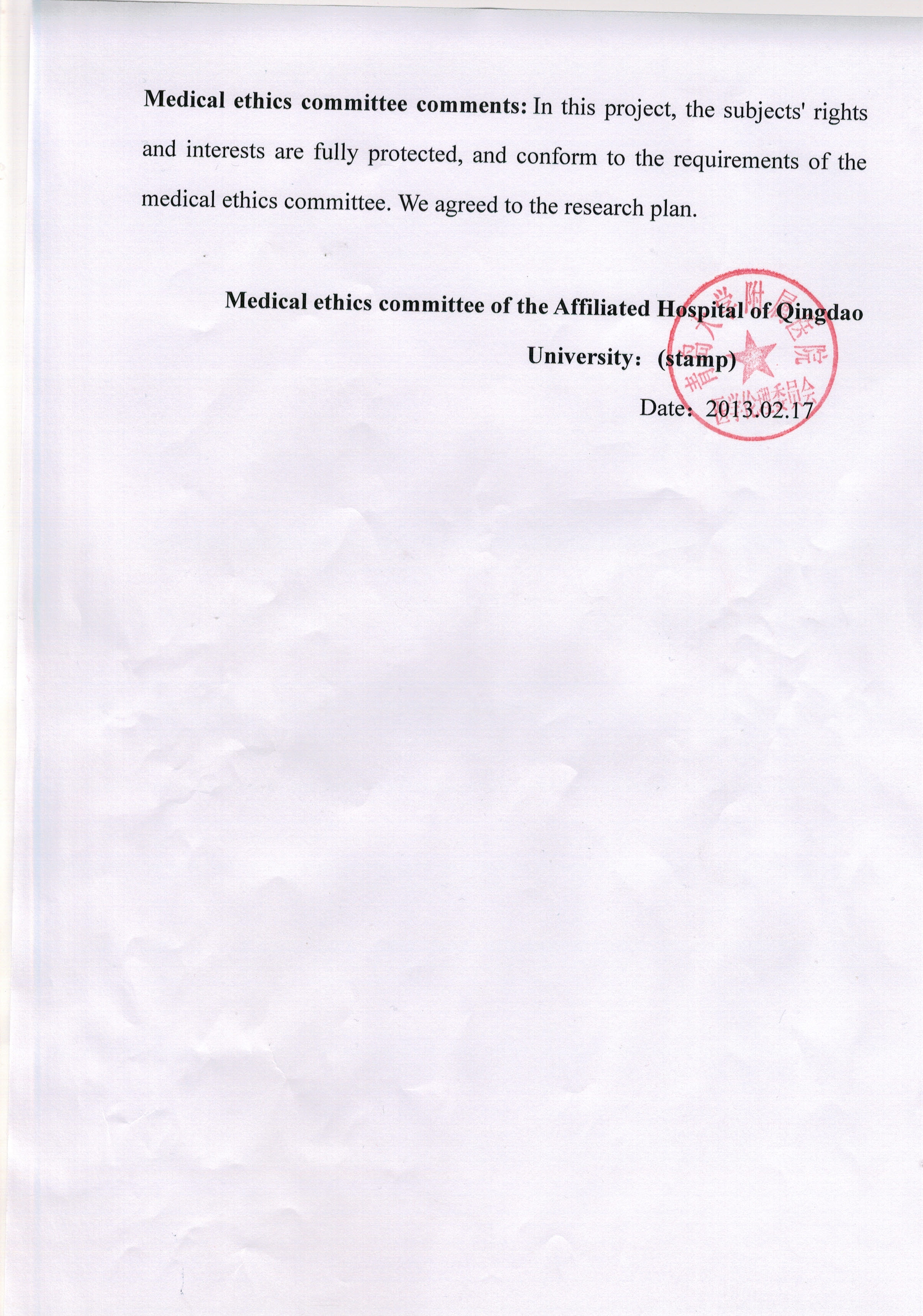

Supplement: S2 Fig — (TIF) [file pone.0174510.s005.tif]
